# The *Salmonella* Type III Effector SspH2 Specifically Exploits the NLR Co-chaperone Activity of SGT1 to Subvert Immunity

**DOI:** 10.1371/journal.ppat.1003518

**Published:** 2013-07-25

**Authors:** Amit P. Bhavsar, Nat F. Brown, Jan Stoepel, Marcel Wiermer, Dale D. O. Martin, Karolynn J. Hsu, Koshi Imami, Colin J. Ross, Michael R. Hayden, Leonard J. Foster, Xin Li, Phil Hieter, B. Brett Finlay

**Affiliations:** 1 Michael Smith Laboratories, The University of British Columbia, Vancouver, British Columbia, Canada; 2 Department of Medical Genetics, The University of British Columbia, Vancouver, British Columbia, Canada; 3 Department of Biochemistry and Molecular Biology, The University of British Columbia, Vancouver, British Columbia, Canada; 4 Centre for High-Throughput Biology, The University of British Columbia, Vancouver, British Columbia, Canada; 5 Department of Microbiology and Immunology, The University of Melbourne, Melbourne, Victoria, Australia; 6 Centre for Molecular Medicine and Therapeutics, The University of British Columbia, Vancouver, British Columbia, Canada; 7 Department of Pediatrics, The University of British Columbia, Vancouver, British Columbia, Canada; 8 Department of Botany, The University of British Columbia, Vancouver, British Columbia, Canada; 9 Department of Microbiology and Immunology, The University of British Columbia, Vancouver, British Columbia, Canada; Osaka University, Japan

## Abstract

To further its pathogenesis, *S*. Typhimurium delivers effector proteins into host cells, including the novel E3 ubiquitin ligase (NEL) effector SspH2. Using model systems in a cross-kingdom approach we gained further insight into the molecular function of this effector. Here, we show that SspH2 modulates innate immunity in both mammalian and plant cells. In mammalian cell culture, SspH2 significantly enhanced Nod1-mediated IL-8 secretion when transiently expressed or bacterially delivered. In addition, SspH2 also enhanced an Rx-dependent hypersensitive response *in planta*. In both of these nucleotide-binding leucine rich repeat receptor (NLR) model systems, SspH2-mediated phenotypes required its catalytic E3 ubiquitin ligase activity and interaction with the conserved host protein SGT1. SGT1 has an essential cell cycle function and an additional function as an NLR co-chaperone in animal and plant cells. Interaction between SspH2 and SGT1 was restricted to SGT1 proteins that have NLR co-chaperone function and accordingly, SspH2 did not affect SGT1 cell cycle functions. Mechanistic studies revealed that SspH2 interacted with, and ubiquitinated Nod1 and could induce Nod1 activity in an agonist-independent manner if catalytically active. Interestingly, SspH2 *in vitro* ubiquitination activity and protein stability were enhanced by SGT1. Overall, this work adds to our understanding of the sophisticated mechanisms used by bacterial effectors to co-opt host pathways by demonstrating that SspH2 can subvert immune responses by selectively exploiting the functions of a conserved host co-chaperone.

## Author Summary

*Salmonella enterica* serovar Typhimurium injects a suite of type III effectors into host cells, but their contributions to pathogenesis remain incompletely understood. We performed cross-kingdom analyses of the *S.* Typhimurium novel E3 ubiquitin ligase (NEL) effector SspH2 using animal, plant and yeast model systems to glean insights into the actions of this effector. We found that SspH2 could discriminately interact with the conserved host protein SGT1, selectively binding human and plant SGT1, which correlates with the evolution of an NLR co-chaperone function in innate immunity in these SGT1 proteins. We found that SspH2 enhanced these SGT1-dependent immune responses in both plant and animal model systems if SspH2 was catalytically active and could interact with SGT1. We also showed that SspH2 interacted with, and modified, the animal NLR Nod1 becoming, to our knowledge, the first effector to directly bind and ubiquitinate this key component of the animal innate immune response. This interaction may underlie the finding that SspH2 activated Nod1 in the absence of its agonist, though this also required SspH2 activity and interaction with SGT1. Intriguingly, SGT1 increased SspH2 protein stability and activity *in vitro* providing an example of a bacterial effector that co-opts the host chaperone machinery to subvert innate immunity.

## Introduction

*Salmonella enterica* serovar Typhimurium (*S.* Typhimurium), a causative agent of gastroenteritis and typhoid-like fever in mammals, encodes two type III secretion systems (T3SS) on *Salmonella* pathogenicity islands (SPI)-1 and SPI-2. T3SSs are proteinaceous channels encoded by Gram negative bacterial pathogens that transport effector proteins directly into infected host cells [1] where they can interface with, and subvert, host cellular processes [2]. SPI-2 effectors are critical for *S.* Typhimurium pathogenesis as they are required for systemic infection [3] although the functions of individual effectors in the host milieu are not fully understood.

Among the suite of effectors delivered by *S.* Typhimurium into the host cell are members of the NEL family of E3 ubiquitin ligases comprised of SspH1, SspH2 and SlrP [4]. This class of E3 ubiquitin ligase has an amino terminal leucine rich repeat (LRR) domain and a carboxy terminal catalytic domain [5]. NEL E3 ubiquitin ligases covalently bind ubiquitin through a conserved catalytic cysteine residue and it is postulated that they are autoinhibited by structural constraint that is relieved upon substrate binding [5]. Host binding partners have been identified and putative functions during pathogenesis assigned to each *S.* Typhimurium NEL effector [4].

We recently identified a novel interaction between SspH2 and the human protein SGT1 (hereafter referred to as HsSGT1) [6]. Originally identified in *Saccharomyces cerevisiae* as an essential cell cycle protein, yeast SGT1 (hereafter referred to as ScSgt1p) interacts with Skp1p, which is a component of the conserved eukaryotic Skp1/Cullin/F-box (SCF) E3 ubiquitin ligase. ScSgt1p is required for progression through both the G1/S and G2/M checkpoints of the cell cycle [7]. Homologs of SGT1 that retain these essential G1 and G2 cell cycle functions have been identified in both animal and plant kingdoms. Intriguingly, mammalian and plant SGT1 orthologs have gained additional functional roles in innate immunity as nucleotide-binding leucine rich repeat receptor (NLR) co-chaperones through their association with HSP90 [8], [9], [10], [11].

In animal innate immunity Nod1 is a canonical NLR. Upon recognition of the bacterial peptidoglycan dipeptide component, iE-DAP, Nod1 is activated and signals through the transcription factor NF-κB to express pro-inflammatory chemokines such as IL-8 [12]. Importantly, HsSGT1 was shown to co-chaperone Nod1 and HsSGT1-silencing abrogates Nod1-mediated IL-8 secretion *in vitro* [10]. In contrast to mammalian NLRs that recognize general microbial structures, plant NLRs recognize pathogen-specific components, and upon activation, can induce programmed cell death as part of the hypersensitive response (HR) [13], [14]. For example, the potato NLR, Rx, recognizes the coat protein of potato virus X (PVX), and subsequently inhibits viral replication or induces an HR to stop viral spread [15]. Several plant NLRs, including Rx, are functionally dependent on SGT1 co-chaperone activity, in conjunction with HSP90 and RAR1 [16]. Model assays have been developed for SGT1 NLR co-chaperone function in both animal and plant systems [10], [17].

In this study we used animal, plant and yeast model systems to gain insights into the biological and mechanistic function of the enigmatic *S.* Typhimurium NEL type III effector, SspH2. We identified a novel functional role for SspH2 in host cells where catalytically active SspH2 enhanced SGT1-dependent NLR-mediated immunity in both animal and plant model systems. SspH2 selectively bound NLR co-chaperone-competent SGT1 and the mammalian NLR, Nod1, in a trimeric complex and monoubiquitinated Nod1. SspH2 did not affect essential SGT1 G1 and G2 cell cycle functions, however SspH2 protein stability and activity were enhanced by SGT1 *in vitro*. Thus, these cross-kingdom investigations have helped define a functional role for *S.* Typhimurium SspH2 that centers on the subversion of immune-specific functions of the host protein SGT1.

## Results

### Characterizing the SspH2/SGT1 interaction

**Figure 1:**
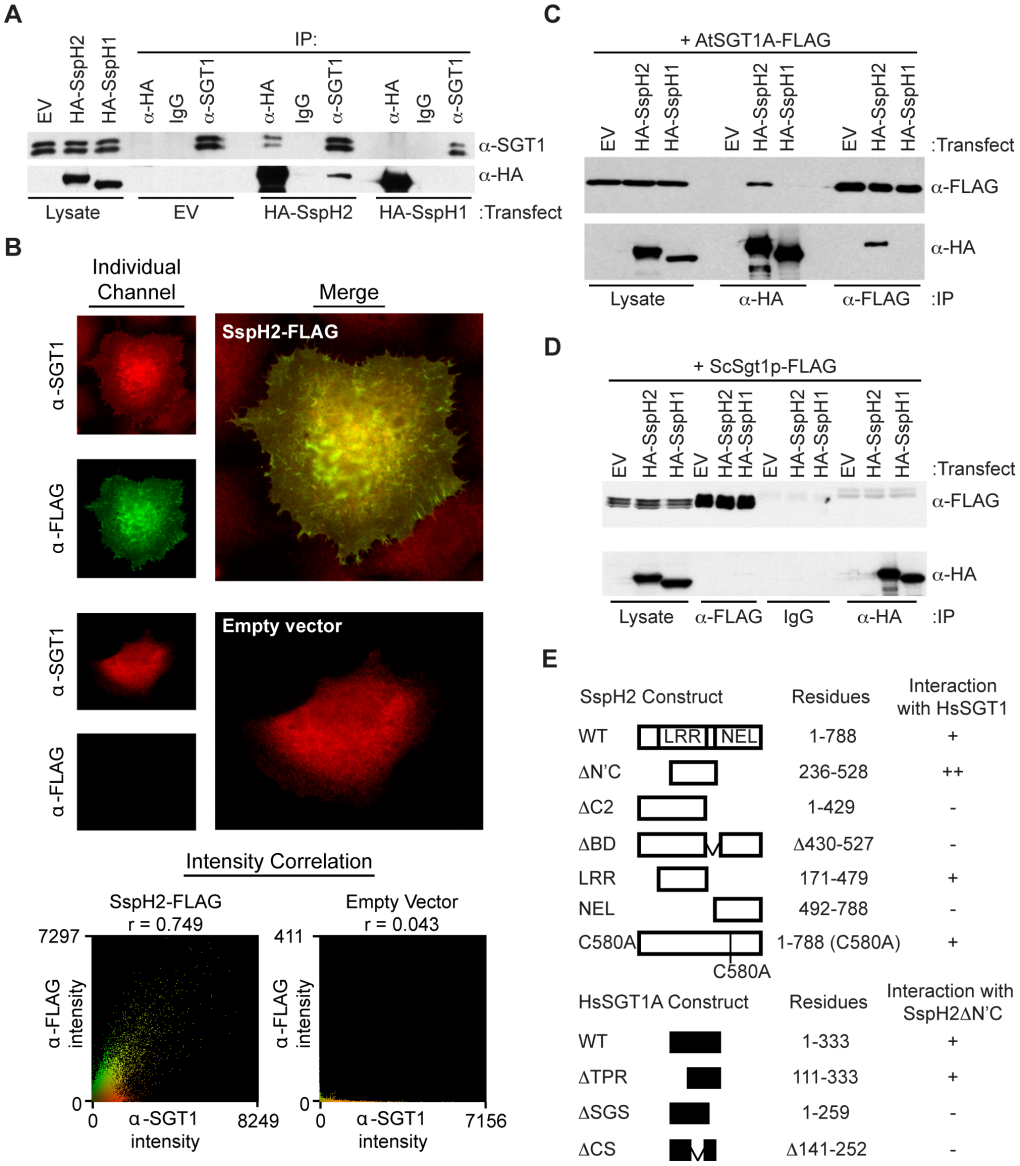
Characterization of the interaction between *S.* Typhimurium SspH2 and cross-kingdom SGT1 proteins. **A**, Reciprocal co-immunoprecipitation (IP) analyses of HsSGT1 and SspH2 or SspH1 transiently expressed in HEK 293T cells. **B**, Co-localization analysis of SspH2-FLAG and HsSGT1. Following immunofluorescence staining stacks were collected, deconvolved and subject to intensity correlation analysis to quantify colocalisation of the anti-FLAG and anti-SGT1 signals. Single slices from representative stacks are shown. The intensity correlation analysis from the whole stack images are shown as scatter plots, together with the respective r values. **C–D**, Reciprocal co- (IP) analyses of AtSGT1A (**C**) or ScSgt1p (**D**) and SspH2 or SspH1 transiently expressed in HEK 293T cells. For IP experiments *S.* Typhimurium effectors and exogenous SGT1 were tagged with tandem hemagglutinin (HA) and FLAG epitopes, respectively. IPs and immunoblotting (IB) were performed with the indicated antibodies. For panels A, C and D, EV denotes the empty vector. **E**, Summary of SspH2 and HsSGT1 interaction studies. Wild-type-, enhanced- and absent or strongly reduced-interaction is indicated by +, ++ and −, respectively. The raw data are shown in Fig. S1A and B.

We sought to characterize the interaction between SspH2 and HsSGT1 and determine the functional consequences of this interaction. The specificity of the interaction was confirmed because SspH2, but not SspH1 that shares approximately 70% sequence identity [4], co-precipitated with HsSGT1 when expressed in human embryonic kidney (HEK) 293T cells (Fig. 1A). SGT1 has three domains, an amino terminal tetratricopeptide repeat (TPR) domain, a central Chord and SGT1 (CS) domain, and a carboxy terminal SGT1-specific (SGS) domain [16]. In humans SGT1 has two isoforms that are splice variants, HsSGT1A and HsSGT1B [16], and both interacted with SspH2 (Fig. 1A). Similarly, when expressed in cell culture, SspH2 co-localized with endogenous HsSGT1 when examined by immunofluorescence (Fig. 1B).

Given the conservation of SGT1 proteins among eukaryotes we tested for interactions between SspH2 and SGT1 proteins from plant (*Arabidopsis thaliana*) and yeast (*S. cerevisiae*) using co-expression in 293T cells. *A. thaliana* also has two SGT1 isoforms, AtSGT1A and AtSGT1B that each share approximately 40% identity/60% similarity with HsSGT1A [16]. Intriguingly, a similar specific interaction was detected between AtSGT1A or AtSGT1B and SspH2, but not SspH1 (Fig. 1C and Fig. S4A). By contrast, SspH2 did not interact with ScSgt1p, which shares only ∼25% identity with HsSGT1A (Fig. 1D).

To further characterize the interaction between SspH2 and SGT1, truncation variants of HsSGT1A and SspH2 were generated and tested in the interaction assay described above (Fig. 1E; primary data is provided in Fig. S1A and S1B). Expression of the SspH2 LRR domain, but not the NEL domain, retained interaction with HsSGT1. An HsSGT1 binding region was identified in the distal portion of the SspH2 LRR domain since a construct lacking residues 430–527 of SspH2 (designated SspH2ΔBD) did not interact with HsSGT1. By contrast, the SspH2 ubiquitin ligase catalytic mutant (C580A) [5] retained interaction with HsSGT1. Similar truncation analyses with HsSGT1 indicated that both the CS and SGS domains of HsSGT1 were required for binding to SspH2 (Fig. 1E).

### SspH2 enhances SGT1-dependent Nod1 activity

**Figure 2:**
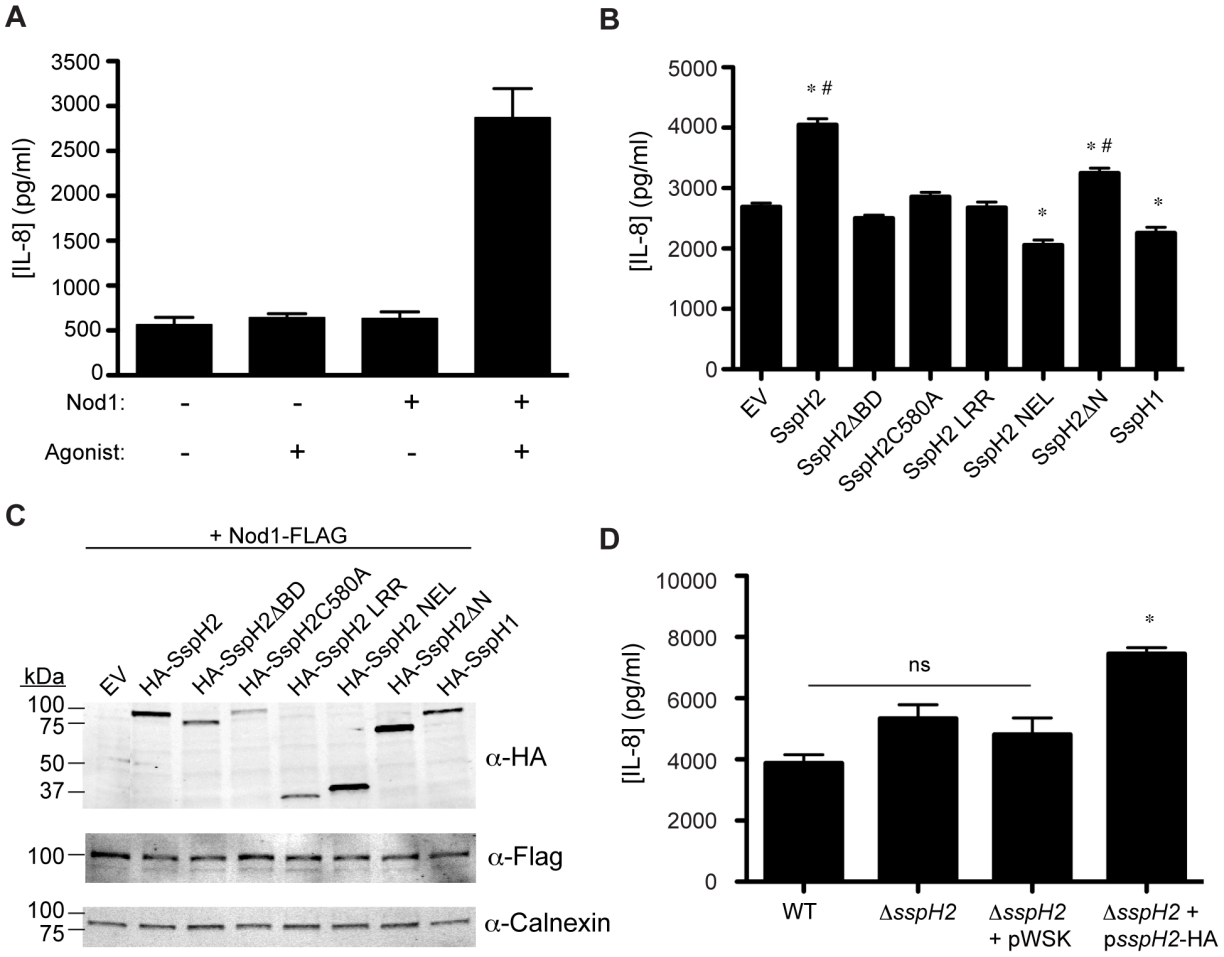
*S.* Typhimurium SspH2 enhances SGT1-dependent NLR immune response in mammalian cells. **A**, Establishment of a Nod1-mediated IL-8 secretion assay in HeLa cells. Secreted IL-8 was quantified in the presence and absence of transfected Nod1 and Nod1 agonist. See Materials and methods for more detail. **B**, Effect of *S.* Typhimurium effector constructs on Nod1-mediated IL-8 secretion. HeLa cells were co-transfected with Nod1 and effector constructs and treated with Nod1 agonist. Data are presented as the mean ± standard error of the mean for three (**A**) and twelve (**B**) independent determinations. Data were analyzed using a non-parametric Mann-Whitney test and * denotes *p*<0.005 between the indicated sample and the empty vector control (EV) sample. # denotes *p*<0.005 between the indicated samples. **C**, The expression of epitope tagged *S.* Typhimurium effectors (HA) and exogenous Nod1 (FLAG) was assessed by immunoblot in total lysate. Calnexin is a loading control. **D**, Effect of *S.* Typhimurium infection on IL-8 secretion. HeLa cells were infected with the wild type strain, *sspH2* deletion mutant (Δ*sspH2*), and *sspH2* deletion mutant complemented with empty vector (Δ*sspH2*+pWSK) or *sspH2* (Δ*sspH2*+p*sspH*2-HA). See Materials and methods for more detail. Data are presented as the mean ± standard error of the mean for six independent determinations. Data were analyzed using a non-parametric Mann-Whitney test and * denotes *p*<0.005 between the indicated sample and all other samples. ns, denotes no significant difference between the indicated samples.

Notably, the CS and SGS domains of HsSGT1 were previously reported to facilitate its co-chaperone function of the mammalian NLR, Nod1, in animal innate immunity [10], [11]. Given that interaction with SspH2 involved the same two HsSGT1 domains, an *in vitro* Nod1-dependent IL-8 secretion assay was developed in HeLa cells (see Fig. 2A and [10]) and used to test the impact of SspH2 on this SGT1-dependent Nod1 response. Unexpectedly, SspH2 expression caused a significant increase in IL-8 secretion (Fig. 2B). SspH2ΔBD and SspH2C580A failed to enhance IL-8 secretion in comparison to wild type SspH2 (Fig. 2B) suggesting that both HsSGT1 binding, as well as a functional ubiquitin ligase domain, are required for this phenotype. Similarly, expression of the SspH2 LRR or NEL domains individually failed to recapitulate the phenotype of enhanced IL-8 secretion induced by full length SspH2 (Fig. 2B). Interestingly, SspH2ΔN, an SspH2 variant that lacks the first 163 amino acids including the palmitoylation site and membrane-targeting sequence recently identified [18] also significantly enhanced IL-8 secretion, albeit not to the same extent as the full-length protein. Lysate from cell culture was analyzed by Western blot to verify expression of all relevant constructs (Fig. 2C). Collectively, these data suggest that SspH2 binds HsSGT1 and stimulates Nod1 through a mechanism that involves ubiquitin transfer, though SspH2 palmitoylation and/or membrane localization is not strictly required. In addition, IL-8 secretion was also measured in HeLa cells infected with *S.* Typhimurium strains containing a deletion of *sspH2*. Infection with the *sspH2* deletion strain complemented with *sspH2*, but not empty vector, showed a significant increase in IL-8 secretion compared to the deletion strain (Fig. 2D). Taken together, these data indicate that SspH2 either expressed directly in host cells, or bacterially delivered, can enhance Nod1 response.

In this work we have used SspH2 expression constructs that are epitope-tagged at the amino or carboxyl terminal. Given the recent identification of a palmitoylation site at the amino terminus of SspH2 [18] we tested to what extent the position of the epitope tag impacted on SspH2 phenotypes. Both amino- and carboxyl-terminal tagged versions of SspH2 showed associations with HsSGT1 via co-immunoprecipitation or co-localization, respectively (Fig. 1A and B). We also verified that amino-terminal tagged SspH2 was palmitoylated in host cells (Fig. S2A) and that the enhancement of Nod1 activity was indistinguishable between both amino- and carboxyl-tagged SspH2 constructs (Fig. S2B). On the basis of these results we conclude that amino- and carboxyl-terminal tagged SspH2 constructs are functionally equivalent.

### SspH2 enhancement of SGT1-dependent NLR activity is similarly conserved in plants

**Figure 3:**
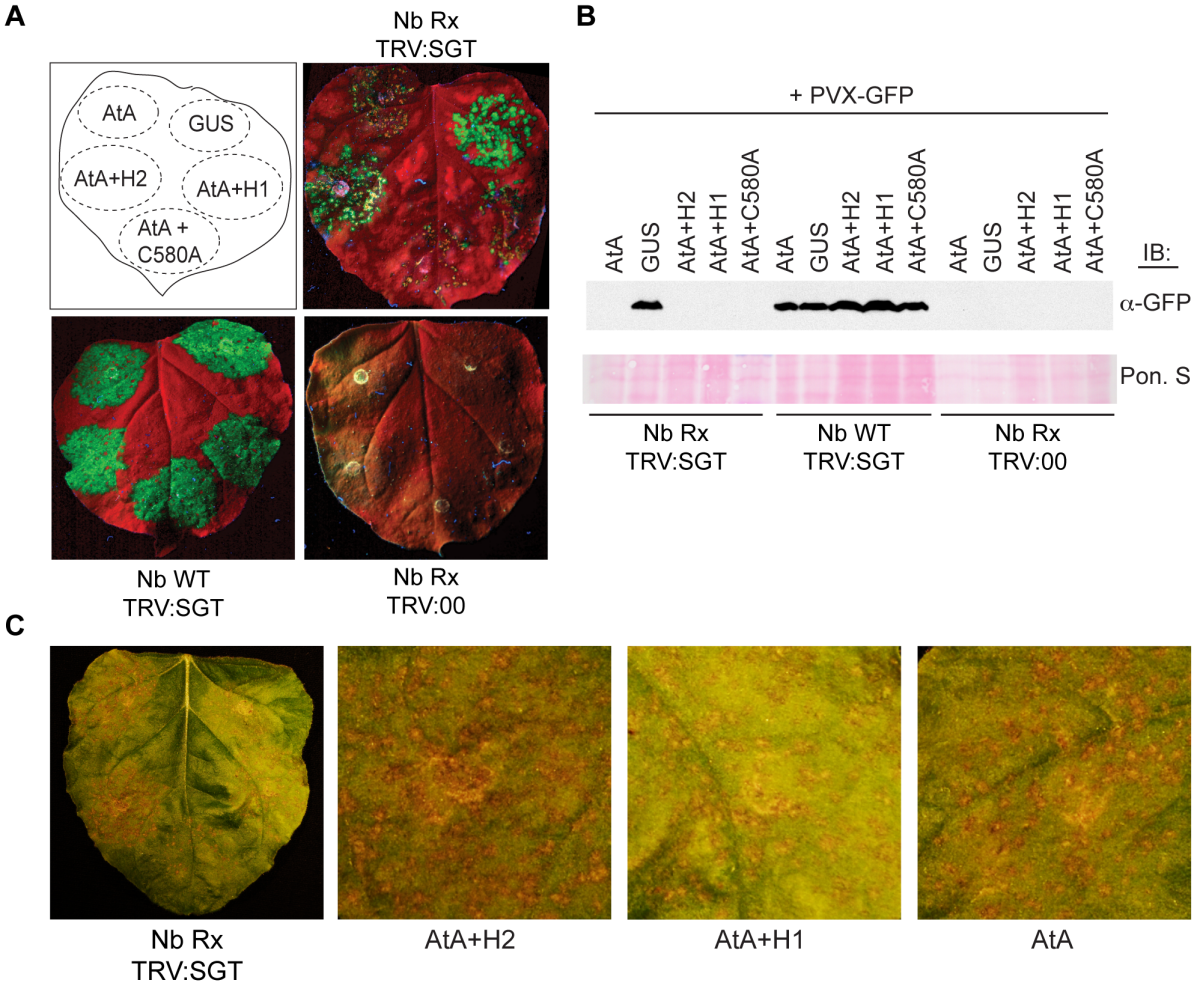
*S.* Typhimurium SspH2 enhances SGT1-dependent NLR immune response *in planta*. **A**, An NbSGT1-silenced (TRV:SGT) Rx-transgenic *N. benthamiana* (Nb Rx) leaf was agro-infiltrated with PVX-GFP and also 5xMyc-AtSGT1A (AtA), GUS, SspH2-3xFLAG (H2), SspH1-3xFLAG (H1) and SspH2C580A-3xFLAG (C580A) as indicated. As controls the same infiltrations were performed in a control-silenced (TRV:00) Nb Rx leaf and non-transgenic (Nb WT) TRV:SGT leaf. Leaves were imaged under UV lighting 7 days post-infiltration. **B**, Infiltration site lysate from samples in (**A**) were immunoblotted with α-GFP to detect PVX replication. Membranes were stained with Ponceau S (Pon. S) to indicate protein loading. **C**, Nb Rx TRV:SGT leaf was agro-infiltrated as indicated in (**A**) and imaged under brightfield 7 days post-infiltration. Higher magnification panels of the brightfield image are provided for comparison.

Since both animal and plant SGT1 proteins are critical NLR co-chaperones, we examined whether SspH2 specifically enhances Nod1 response, or whether SspH2 generally alters SGT1-dependent immune responses. Accordingly, the impact of SspH2 was tested using the established SGT1-dependent Rx/PVX immunity assay *in planta* [19]. This assay employs a PVX variant with genomically encoded GFP to readily visualize PVX replication and has been reconstituted in transgenic Rx-expressing tobacco (*Nicotiana benthamiana*) to facilitate transient gene silencing and expression [19]. A schematic depiction of the assay that outlines the procedure, experimental and control constructs, and the functional readouts from the assay is shown in Figure S3. Endogenous *N. benthamiana* SGT1 (NbSGT1) was silenced [19] and complemented with AtSGT1A because its co-chaperone activity confers Rx stability (thereby mediating resistance to PVX [20]) and because of its robust interaction with SspH2 (Fig. 1C). As controls for the Rx/PVX immunity assay we verified (*i*) that NbSGT1 depletion was required for PVX replication in Rx-transgenic *N. benthamiana* (Fig. 3A; compare Nb Rx TRV:SGT and Nb Rx TRV:00), (*ii*) that immune responses mounted to PVX were dependent on Rx (Fig. 3A; compare Nb Rx TRV:SGT and Nb WT TRV:SGT) and (*iii*) that all constructs expressed appropriately (Fig. S4B).

As expected in the Rx/PVX immunity assay, complementation of NbSGT1 with β-glucuronidase (GUS) as a negative control resulted in strong fluorescence (Fig. 3A). The fluorescent signal was attributed to elevated PVX-GFP levels since lysate from this infiltration contained readily detectable GFP (Fig. 3B). These data verify that PVX replication is robust in the absence of SGT1. Interestingly, co-expression of AtSGT1A and SspH2, but not SspH1 or SspH2C580A, also yielded a strong fluorescent signal that was observed in approximately 70% of leaves (Fig. 3A). However, GFP levels were undetectable in this infiltration site (Fig. 3B) indicating that PVX replication was still suppressed in the AtSGT1A+SspH2 infiltration. This suggested that the observed phenotype might be caused by autofluorescence, which has been previously described as a marker of the HR immune response [21].

To assess the effect of SspH2 on HR in the transgenic *N. benthamiana* assay, infiltrations were performed as outlined above and the formation of necrotic-like lesions was examined. SspH2, but not SspH1 or SspH2C580A, elicited enhanced HR in comparison to AtSGT1A expression alone (Fig. 3C). Importantly, co-expression of SspH2, AtSGT1A and Rx, did not elicit HR lesions in the absence of PVX (Fig. S4C), indicating that SspH2 toxicity was not contributing to HR development. Similarly, co-expression of SspH2, Rx and PVX did not elicit HR lesions in the absence of AtSGT1A, confirming the SGT1-dependence of the phenotype (Fig. S4C). These data indicate that active SspH2 interacts with AtSGT1A to enhance plant innate immunity, and taken together with the Nod1 data, implies that SspH2 utilizes a conserved mechanism to alter SGT1-dependent immune responses across kingdoms.

### SspH2 interacts with NLR co-chaperone-competent SGT1

**Figure 4:**
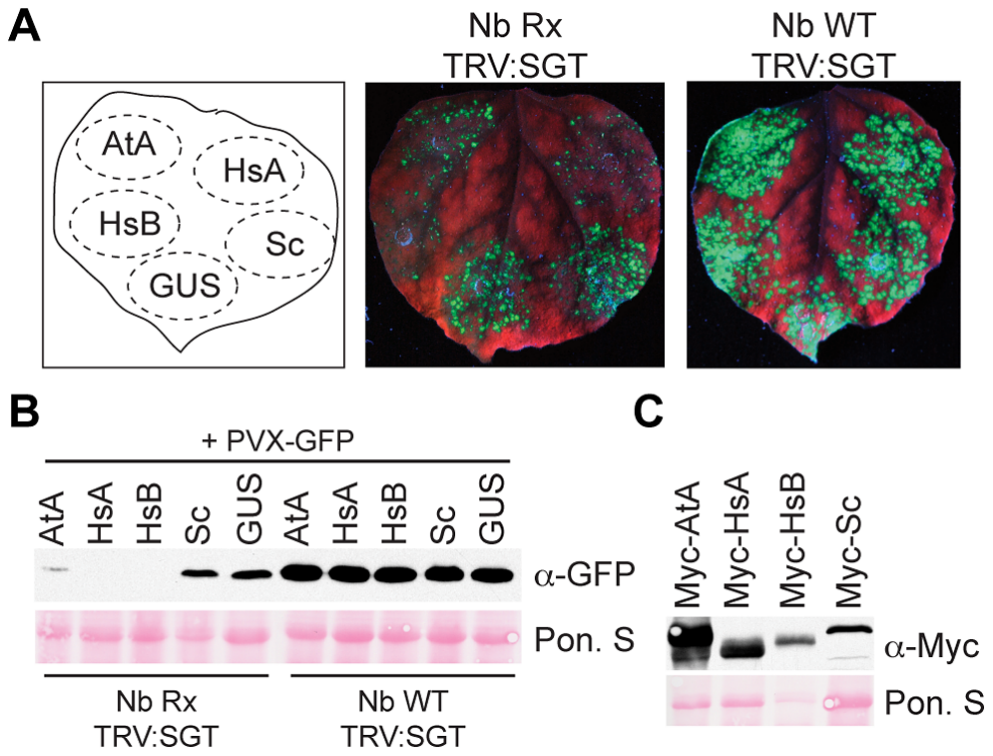
Yeast ScSgt1p cannot function as an NLR co-chaperone. **A,** Nb Rx TRV:SGT or Nb WT TRV:SGT leaves were agro-infiltrated with PVX-GFP and also Myc epitope tagged AtSGT1A (AtA), HsSGT1A (HsA), HsSGT1B (HsB), ScSgt1p (Sc) and GUS as indicated. Leaves were imaged under UV lighting 7 days post-infiltration. **B,** Infiltration site lysate was immunoblotted with α-GFP to detect PVX replication. **C,** Whole leaf lysate was immunoblotted with α-Myc to detect SGT1 construct expression. Membranes were stained with Ponceau S (Pon. S) to indicate protein loading.

Our data indicates that SspH2 interacts with SGT1 proteins from human and plant, but not yeast. Since both human and plant SGT1 proteins complement ScSgt1p cell cycle mutants in yeast [7], [9] we hypothesized that the basis for SspH2 and SGT1 interaction is the evolution of NLR co-chaperone function in human and plant SGT1. Accordingly, SGT1 proteins from plant, human and yeast were assayed for Rx co-chaperone function *in planta*. HsSGT1A/B and AtSGT1A functionally restored Rx-mediated PVX immunity *in planta* as indicated by diminished fluorescence (Fig. 4A) and little detectable GFP in these infiltration sites (Fig. 4B). By contrast, fluorescence and GFP levels were virtually indistinguishable between ScSgt1p and the GUS negative control (Fig. 4A and B) confirming that ScSgt1p does not function as an NLR co-chaperone *in planta*. Whole leaf infiltrations verified expression of all SGT1 constructs (Fig. 4C). These data support the hypothesis that SspH2 specifically exploits SGT1 NLR co-chaperone functional determinants for binding.

### SspH2 does not alter the G1 and G2 cell cycle functions of SGT1

**Figure 5:**
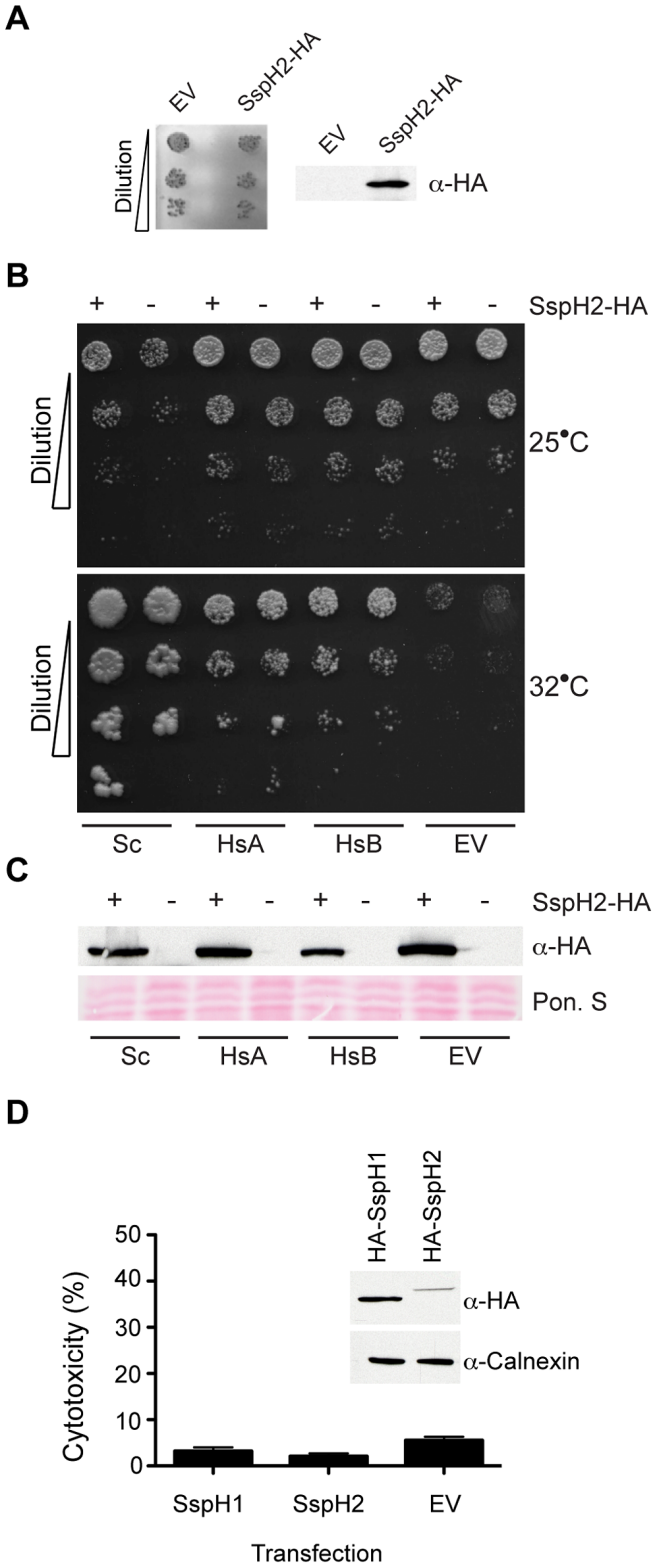
*S.* Typhimurium SspH2 does not alter the essential cell cycle function of SGT1. **A,** Dilution series of wild type yeast expressing empty vector (EV) or SspH2 with HA-epitope tag grown at 25°C. The expression of HA-tagged SspH2 was assessed by immunoblot in total lysate. **B,** Dilution series of yeast *sgt1-3* complemented with ScSgt1p (Sc), HsSGT1A (HsA), HsSGT1B (HsB) or empty vector (EV) and expressing SspH2-HA (+) or empty vector (−), were grown at the permissive (25°C) and restrictive (32°C) temperatures. **C,** The expression of HA-tagged SspH2 in cultures from (**B**) was assessed by immunoblot in total cell lysate. The membrane was stained with Ponceau S (Pon. S) to indicate protein loading. **D,** HeLa cells transfected with plasmid control (EV) or constitutive expression vectors for SspH1 or SspH2 were quantified for cytotoxicity. Data are presented as the mean ± standard error of the mean for three independent determinations. *S.* Typhimurium effectors were tagged with an HA epitope and shown in the inset is the expression of transfected constructs as assessed by α-HA IB. Calnexin is a loading control.

Having shown that SspH2 enhances SGT1-dependent NLR signaling in both mammalian and plant model systems, we next examined if SspH2 altered SGT1-dependent cell cycle progression. Yeast provides a robust model with which to study the essential cell cycle function of SGT1 and accordingly SspH2 was expressed in yeast with no apparent affect on cell viability (Fig. 5A). The absence of an SspH2 phenotype in this assay might have been due to the lack of interaction between SspH2 and ScSgt1p (Fig. 1D). Therefore, we re-examined the impact of SspH2 on SGT1 G2 cell cycle function using a characterized ScSgt1p temperature sensitive yeast mutant (*sgt1-3*) that, unless complemented by a functional SGT1 allele [7], arrests in G2 resulting in cell death when grown at the restrictive temperature. Expression of either HsSGT1A or HsSGT1B rescued yeast viability of the *sgt1-3* mutant at the non-permissive temperature, and this complementation was unaffected by the presence or absence of SspH2 (Fig. 5B and C) indicating that SspH2 does not impinge upon the G2 cell cycle function of SGT1. Similar results were also obtained with the *sgt1-5* yeast mutant strain that arrests in the G1 phase of the cell cycle at restrictive temperatures (data not shown).

Nevertheless, the yeast *sgt1-3* complementation assay has some limitations that warrant caution in the interpretation of these results. For example, the overexpression of human SGT1 might mask any potential SspH2-mediated inhibition of SGT1-cell cycle function. To address this issue SspH2 was transiently transfected into mammalian cultured cells expressing endogenous levels of human SGT1. We reasoned that if SspH2 impinged on the essential cell cycle function of SGT1 this would manifest as an increased cytotoxic response because SGT1 silencing leads to cell death in mammalian cultured cells [22]. Consistent with the yeast *sgt1-3* complementation assay, SspH2 expression in cultured mammalian cells showed no impairment in this assay compared to the empty vector control (Fig. 5C). Thus, these data suggest that SspH2 discriminates between the cell cycle and NLR co-chaperone functions of SGT1, selectively targeting the latter through interactions dictated by the functional diversification of SGT1 as an NLR co-chaperone.

### SspH2 interacts with Nod1 and causes agonist-independent Nod1 activation

**Figure 6:**
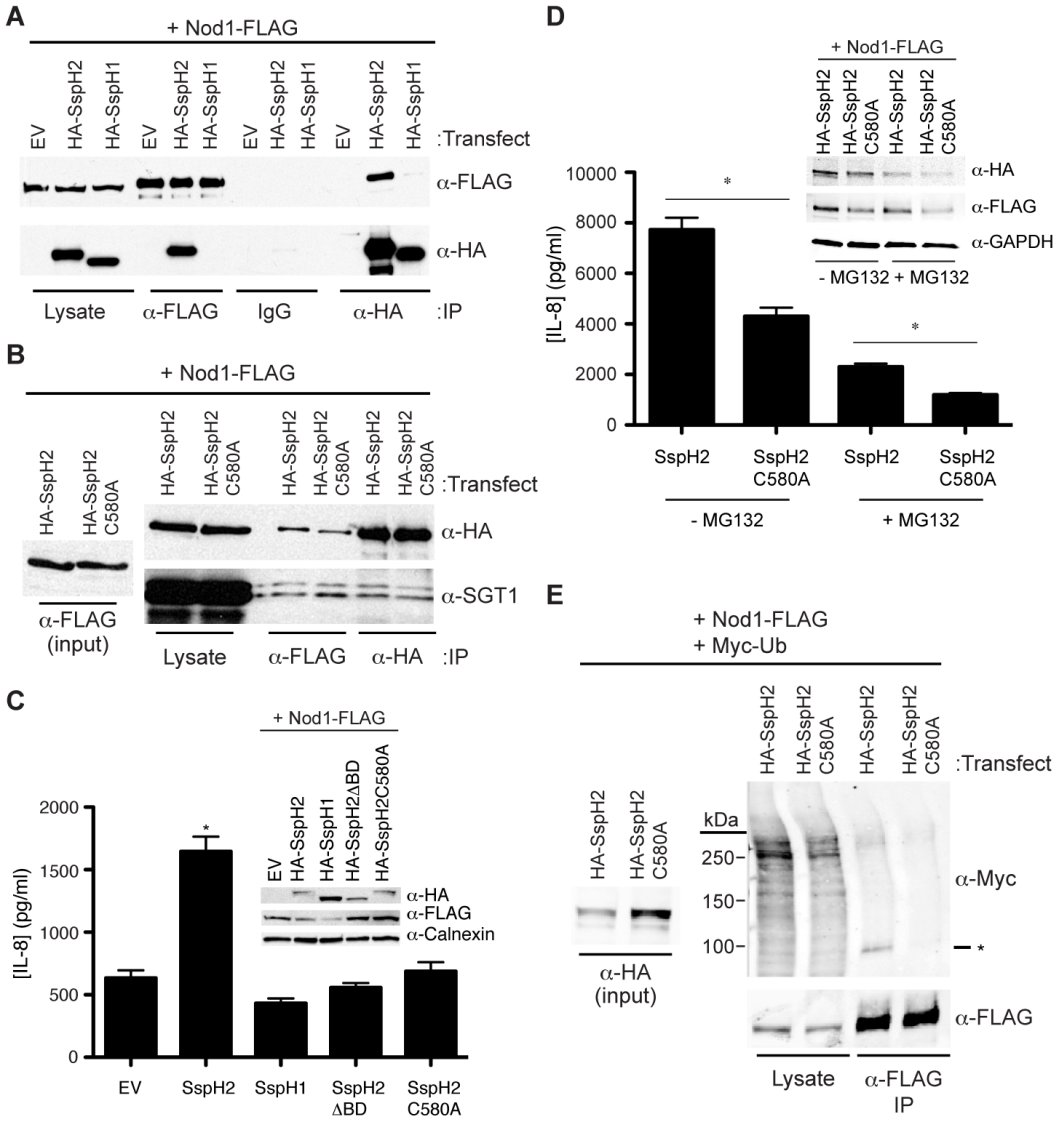
*S.* Typhimurium SspH2 interacts with and ubiquitinates Nod1 causing agonist-independent activation of Nod1. **A,** Reciprocal co-IP analyses of Nod1 and SspH2 or SspH1 transiently expressed in HEK 293T cells. *S.* Typhimurium effectors and Nod1 were tagged with HA and FLAG epitopes, respectively. IPs and immunoblotting (IB) were performed with the indicated antibodies. **B,** Co-IP analysis of transiently expressed Nod1 and either SspH2 or SspH2C580A and endogenous SGT1 in HEK 293T cells. SspH2 constructs and Nod1 were tagged with HA and FLAG epitopes, respectively. IPs and IB were performed with the indicated antibodies. **C,** Effect of *S.* Typhimurium effector constructs on Nod1-mediated IL-8 secretion. HeLa cells were co-transfected with Nod1 and effector constructs but not treated with Nod1 agonist. Data are presented as the mean ± standard error of the mean for three independent determinations. Data were analyzed using a non-parametric Mann-Whitney test and * denotes *p*<0.005 between the SspH2 and empty vector control (EV) samples. *S.* Typhimurium effectors and Nod1 were tagged with HA and FLAG epitopes, respectively. Shown in the inset is the expression of transfected constructs as assessed by IB with the indicated antibodies. Calnexin is a loading control. **D,** Effect of proteasome inhibitor on SspH2-mediated enhancement of IL-8 secretion. HeLa cells were co-transfected with Nod1 and SspH2 or SspH2C580A and treated with MG132 or vehicle during Nod1 agonist addition. Data are presented as the mean ± standard error of the mean for eight independent determinations. Data were analyzed using a non-parametric Mann-Whitney test and * denotes *p*<0.0005 between the SspH2 and SspH2C580A control samples. SspH2 constructs and Nod1 were tagged with HA and FLAG epitopes, respectively. Shown in the inset is the expression of transfected constructs as assessed by IB with the indicated antibodies. GAPDH is a loading control. **E,** Co-IP analyses of Nod1, ubiquitin and SspH2 or SspH2C580A transiently expressed in HEK 293T cells. SspH2 constructs, Nod1 and ubiquitin were tagged with HA, FLAG and Myc epitopes, respectively. IPs and IB were performed with the indicated antibodies. The α-Flag IP samples were co-immunoblotted against α-Flag and α-Myc antibodies and detected in separate channels, confirming that the Nod1 and ubiquitin signals overlaid at the indicated band (*).

Having observed that SspH2 phenotypes were restricted to the enhancement of SGT1-dependent immune responses we sought to further characterize this phenotype in the Nod1 assay. SGT1 has been reported to bind Nod1 [10], [11] and given that SspH2 also interacts with SGT1 we examined if SspH2 could interact with Nod1. We observed that SspH2, but not SspH1, could specifically interact with Nod1 via reciprocal co-immunoprecipitation in cell culture lysate (Fig. 6A). Further characterization of this interaction identified a putative ternary complex between SspH2, Nod1 and SGT1 in cultured mammalian cells (Fig. 6B). SspH2 E3 ubiquitin ligase activity was not required for its interaction with Nod1 since SspH2C580A also co-immunoprecipitated with Nod1 and SGT1 (Fig. 6B).

Having identified a putative ternary complex between SspH2, Nod1 and SGT1 we sought to functionally characterize these interactions. We postulated that this interaction might be the basis for the SspH2-dependent activation of Nod1, perhaps through the induction of a conformational change in Nod1. Accordingly, Nod1 activity assays were repeated in the absence of the Nod1 agonist (iE-DAP/hIFN-γ). Intriguingly, even in the absence of agonist, catalytically active SspH2 induced a significant increase in IL-8 secretion in the assay (Fig. 6C). SGT1 was again implicated in this SspH2 phenotype, since SspH2ΔBD did not significantly enhance IL-8 secretion.

To study the role of SspH2 catalytic activity on Nod1 activation, IL-8 secretion assays were performed in the presence of the proteasome inhibitor MG132. Despite a marked decrease in IL-8 secretion, the presence of MG132 did not abrogate the significant increase in IL-8 secretion elicited by SspH2 compared to SspH2C580A (Figure 6D). These data suggest that the proteasomal degradation of an SspH2 substrate(s) does not contribute to the enhancement of Nod1 activity. To further characterize the requirement of SspH2 catalytic activity in the enhanced NLR response phenotype, mammalian cultured cells were transiently co-transfected with epitope-tagged SspH2, Nod1 and ubiquitin constructs, followed by Nod1 immunoprecipitation. Notably, transfection of catalytically active SspH2, but not SspH2C580A, resulted in the apparent ubiquitination of Nod1 (Fig. 6E). Dual detection of Nod1 and ubiquitin in the Nod1-immunoprecipitate indicated co-migration of the major ubiquitin and Nod1 signals (indicated by an asterisk in Fig. 6E). The apparent molecular weight of the Nod1-ubiquitin species suggests that SspH2 monoubiquitinates Nod1.

### Interaction with SGT1 enhances SspH2 stability and activity *in vitro*

**Figure 7:**
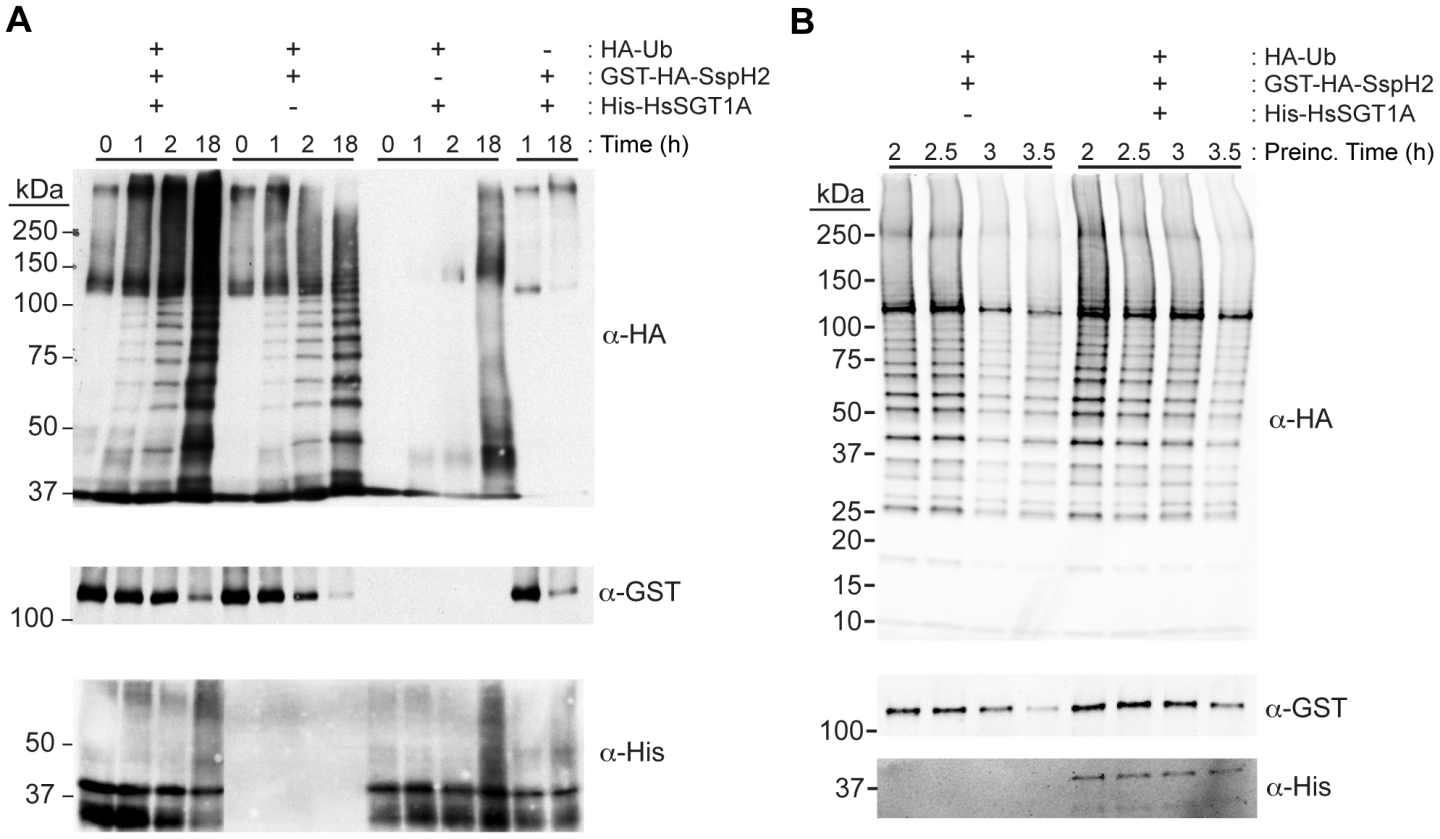
SspH2 stability and activity are enhanced by HsSGT1A *in vitro*. **A,** Time course of *in vitro* ubiquitination assay conducted with purified recombinant HA-Ubiquitin, UBE1 (E1), UbcH5b (E2), His-HsSGT1A and GST-HA-SspH2 as indicated. Reactions were quenched at the indicated time and immunoblotted with the indicated antibodies to detect SspH2 activity, SspH2 stability and HsSGT1A ubiquitination. **B,** *In vitro* ubiquitination assay reaction components were pre-incubated for the indicated times, initiated with HA-ubiquitin and quenched after 2 hours. Samples were immunoblotted as outlined above. Molecular weights are indicated on the left.

The results obtained above indicated that SspH2 required interaction with co-chaperone-competent SGT1 proteins to enhance NLR responses in both plant and animal systems. However, the nature of the functional interaction between SspH2 and SGT1 remained unclear. Given that Nod1 appeared to be ubiquitinated by SspH2 we tested if SGT1 might also be an SspH2 substrate using recombinant proteins in an *in vitro* ubiquitination assay. SspH2 was catalytically active in this assay with SspH2-dependent ubiquitin polymerization evident after one hour (Fig. 7A). Interestingly, SspH2 E3 ubiquitin ligase activity appeared to be enhanced in the presence of HsSGT1A over the course of the assay as indicated by the intensity of the ubiquitin signal. The enhanced activity was most likely due to increased stability of SspH2 in the presence of HsSGT1A (Fig. 7A). Importantly, SspH2-dependent ubiquitination of HsSGT1A was not observed (Fig. 7A) suggesting that HsSGT1A is not a substrate of SspH2. To further study the apparent stabilization of SspH2 by HsSGT1A, reaction components were pre-incubated together with and without HsSGT1A and initiated by the addition of ubiquitin. Pre-incubation of reaction components in the presence of HsSGT1A showed both increased amounts of SspH2 protein, and increased ubiquitin signal intensity (Fig. 7B). These data are consistent with a model where SspH2 interacts with the host co-chaperone SGT1 in order to stabilize its active conformation. Since SspH2 NEL activity is required for subversion of the immune responses in our assays, these *in vitro* results offer mechanistic insights into the SGT1-dependence of SspH2 phenotypes.

## Discussion

In this study we have uncovered novel aspects of bacterial effector biology through cross-kingdom analyses of model systems. We report that the *S.* Typhimurium E3 ubiquitin ligase SspH2 enhances SGT1-dependent innate immune responses in both animals and plants. Our data show a strong physical and genetic interaction between SspH2 and SGT1 that is required for SspH2-mediated modulation of innate immune responses. Furthermore, biochemical assay suggests that the interaction between these proteins increases SspH2 stability and E3 ubiquitin ligase activity. Interaction with SspH2 is mediated by the SGT1 CS and SGS domains, where the CS domain mediates SGT1 co-chaperone function through interaction with Hsp90 and the SGS domain mediates substrate specificity by binding LRR domain-containing proteins, such as NLRs [23]. This suggests that the LRR domain of SspH2, but not SspH1 or SlrP, may have specifically evolved to mimic an SGT1 client. By contrast, the SGT1 TPR domain, which is dispensable for interaction with SspH2, has been shown to mediate kinetechore assembly and allow progression through the G2/M checkpoint of the yeast cell cycle [16]. Notably, in the assays tested here, SspH2 did not affect SGT1-dependent cell cycle functions in yeast or mammalian cells.

The host SGT1/Hsp90 chaperone machinery represents a unique target for subversion by a bacterial effector. This conserved machinery represents one of the only protein networks that is common to the convergently evolved plant and animal innate immune systems [16], [24]. Previously it was reported that the plant pathogen *Pseudomonas syringae* effector AvrB weakly interacted with AtSGT1B, though AvrB function was mediated by the plant-specific co-chaperone RAR1 [25]. Another *P. syringae* effector, AvrPtoB, showed a genetic, but not physical, interaction with SGT1 and RAR1, requiring these co-chaperones to suppress plant immunity [26]. In plants, HSP70 has been reported to interact with the SGT1/HSP90 chaperone complex and participate in immune signaling [27]. Yet another *P. syringae* effector, HopI1, requires interaction with plant HSP70 to suppress plant immunity presumably by altering HSP70 chaperone dynamics [28]. By contrast, the SspH2 interaction with SGT1 appears to alter the dynamics of effector stability and activity. Since SspH2 catalytic activity is required for its modulation of SGT1-dependent NLR responses, this co-option of the host chaperone machinery may represent a novel mechanism of effector action.

Here we report an intriguing functional link between SspH2 and the enhancement of SGT1-dependent innate immunity in animals and plants. In mammalian cell culture, expression of SspH2 significantly increased Nod1-dependent IL-8 secretion. Similar results were found in bacterial infection studies using a complemented *sspH2* deletion strain. In that experiment no significant difference in IL-8 secretion was detected between the *sspH2* deletion mutant and wild type strain. However, the amount of translocated SspH2 required to elicit the phenotype is unknown and it is possible that this threshold amount is not translocated by the wild type strain. Indeed, it was reported that *sspH2* expression in *S.* Typhimurium was categorized as low when expression of SPI-2 effectors was stratified into very high, high, medium and low categories [29].

*In planta* SspH2 expression caused enhanced intrinsic HR induced by the SGT1-dependent NLR, Rx. Though unusual that an effector would enhance cell death, it was reported very recently that the *Xanthomonas campestris* effector, XopL, also an LRR-containing E3 ubiquitin ligase, causes activity-dependent cell death *in planta* [30]. It is now becoming appreciated that the induction of specific immune responses by bacterial effectors can be beneficial for pathogenic microbes in both plant and animal systems, a concept recently termed ‘effector triggered immune pathology’ (ETIP) [31]. In this context the interaction between SspH2 and Nod1 may well represent the first example of direct recognition of an effector in an animal host.

ETIP blurs the conventional paradigms of host-pathogen interactions. SspH2 targets SGT1 and induces an immune response from an SGT1-dependent NLR in both plant and animal model systems. Similarly, it was recently reported that the *P. syringae* effector AvrRps4 targets the *A. thaliana* immune regulator EDS1 and induces an immune response from the EDS1-dependent NLR, RPS4 [32], [33]. In the RPS4/EDS1/AvrRps4 pathosystem this was suggested to be the mechanism by which RPS4 recognizes and mounts an immune response to the effector AvrRps4. This may also be the case with the SspH2/SGT1/Nod1 pathosystem. Alternatively, SspH2 might exploit SGT1 and its interaction with NLRs to specifically induce ETIP. Particularly fascinating is the discovery that SspH2 can induce SGT1-dependent NLR responses across kingdoms. To our knowledge, SspH2 constitutes the first reported bacterial effector to modulate innate immunity in both plant and animal systems.

Based upon our findings we speculate that SspH2 enhances Nod1 activity through the interaction and modification of this SGT1-dependent NLR. We identified the formation of a trimeric complex of SspH2, Nod1 and SGT1 in immunoprecipitates from cultured mammalian cells; however, the role of SGT1 in complex formation should be further studied since both SspH2 and Nod1 interact with the same SGT1 domains [10], [11]. Catalytically active SspH2 mediated ubiquitination of Nod1, with the apparent molecular weight suggesting that one ubiquitin molecule was transferred to Nod1. Monoubiquitination has been shown to impact cellular processes and signaling, rather than causing proteosomal degradation [34] and this is consistent with our finding that the SspH2-mediated enhancement of Nod1 activity is not altered by proteasomal inhibition. To our knowledge, ubiquitination of Nod1 has not been previously reported and this work sets the stage for further research aimed at uncovering the mechanisms underlying Nod1 ubiquitination and its agonist-independent activation.

## Materials and Methods

### Tissue culture, immunoprecipitations and Nod1 assays

HEK 293T and HeLa cells were cultured in DMEM containing 10% FBS, 1% GlutaMAX, and 1% non-essential amino acids (Life Technologies) at 37°C and 5% CO_2_. For immunoprecipitations HEK 293T cells were seeded at 10^6^ cells per 10 cm dish and transfected the next day using 8–12 µg of Ca_3_(PO_4_)_2_-complexed DNA. Immunoprecipitations were performed as outlined in [6]. For Nod1 activity assays 6×10^4^ cells were seeded per well of a 24-well dish. Cells were transfected using Fugene HD or XtremeGENE 9 (Roche) with a total of 1 µg of DNA [50 ng pcDNA3-Nod1-FLAG (kindly provided by Dana Philpott, University of Toronto), 100–300 ng of effector construct and pcDNA3.1 carrier DNA] and stimulated overnight with Nod1 agonist [1 µg/ml C12-iE-DAP (InvivoGen)+10 ng/ml human interferon gamma (AbD serotec)] in DMEM supplemented with 0.5% FBS on subsequent days. Supernatants were quantified using a human IL-8 ELISA Kit (BD Bioscience). For proteasome inhibition experiments 10 µM MG-132 (or DMSO vehicle) was co-administered with Nod1 agonist. Cell lysate was analyzed with anti-HA and anti-FLAG antibodies to confirm expression of effectors and Nod1, respectively. Cell toxicity was measured using an LDH Assay Kit.

### Immunofluorescence

HeLa cells for immunofluorescence were plated on sterile 12 mm diameter number 1.5 coverslips in 24 well plates at 5×10^4^ cells per well. Forty hours post-transfection cells were fixed for 10 minutes at 37°C in formaldehyde from a 2.5% (w/v) paraformaldehyde solution prior to permeabilisation and blocking in 0.1% (v/v) Triton-X 100, 10% (v/v) normal goat serum. Rabbit anti-flag and mouse anti-SGT1 were each used at 1∶500 and alexa-fluor 488 labelled anti-rabbit and alexa-fluor 568 labelled anti-mouse were used at 1∶250. Stacks were collected at 0.2 µm intervals using an Olympus water-immersion N.A. 1.2 objective and a Photometrics Coolpix HQ_2_ camera, using identical exposure times and gain settings. Stacks were processed by iterative deconvolution using a theoretical point spread function, followed by rolling ball background subtraction and intensity correlation analysis to determine colocalisation [35].

### Bacterial infections of HeLa cells for IL-8 assays

HeLa cells were plated at 1×10^5^ cells per well in 24 well plates the day prior to infection. The *S.* Typhimurium SL1344 *sspH2* deletion strain has been described elsewhere [36]. The *sspH2* deletion strain was transformed with pWSK29 [37] or p*sspH2*-HA.1 (see Text S1 for plasmid construction) for complementation. HeLa cells were infected with SPI-1 induced, invasive *Salmonella* as described previously [38] with the following exceptions: DMEM containing 0.5% (v/v) fetal bovine serum was the medium used during the infection, and the media was changed at 6 h post-infection in order to limit SPI-1-mediated IL-8 secretion. Culture supernatants were collected at 16 h post-infection and stored at −80°C until quantified by ELISA.

### Agro-infiltration, VIGS and PVX assay in *N. benthamiana*

*Agrobacterium tumefaciens* C58C1 containing the pCH32 helper plasmid and pBIN derivatives were grown overnight in YEB medium, harvested and induced overnight in 10 mM MgCl_2_, 1 mM MES, pH 5.6 and 0.1 mM acetosyringone (Sigma). Virus-induced gene silencing (VIGS) of *NbSGT1* was performed on 2–3 week old plants as outlined in [19]. PVX assays were conducted in 3-week VIGS-treated *N. benthamiana* by agro-infiltration at an OD_600_ of 1 comprised of SGT1-, effector- and PVX-GFP-expressing strains at an OD_600_ of 0.3, 0.6 and 0.001, respectively, and pBIN61 containing strain as carrier. pBIN61-*GUS* expressing strain was used as a negative control. VIGS-, AtSGT1-, and GUS-constructs were kindly provided by Ken Shirasu (RIKEN). Agro-infiltrated *N. benthamiana* was grown for approximately 7 days. PVX data are representative of three or more independent experiments. Leaf lysate was analyzed with anti-GFP, anti-Myc and anti-FLAG antibodies to confirm expression of PVX, SGT1 and effector constructs, respectively.

### Ubiquitination assay

*In vitro* ubiquitination reactions (60 µl) using purified recombinant GST-SspH2 (1 µg), His-HsSGT1A (1 µg), HA-Ubiquitin (1.5 µg), UBE1 (0.4 µg), UbcH5b (1.5 µg) (BostonBiochem) were performed as in [5]. For pre-incubation experiments GST-SspH2 (.4 µg) was pre-incubated with all reaction components as indicated above (except ubiquitin) at 37°C. Reactions were initiated by the addition of HA-Ubiquitin. The reaction buffer was: 80 mM Tris pH 7.5, 50 mM NaCl, 10 mM MgCl_2_, 5 mM ATP, 0.1 mM DTT. Reaction aliquots were quenched with SDS-PAGE loading buffer. Samples were immunoblotted as outlined above. For Nod1 ubiquitination studies 293T cells were seeded at 4×10^5^ cells/well of a 6-well dish. The following day cells were transfected using XtremeGENE 9 reagent (Roche) according to the manufacturer's specifications with (per well) 200 ng Myc-ubiquitin construct, 200 or 400 ng Nod1-FLAG construct, 100 or 200 ng HA-SspH2 or HA-SspH2C580A construct, and empty vector carrier DNA to 1 µg total DNA. Cell lysate was harvested after 48 h and was immunoprecipitated with α-FLAG antibody as outlined above.

### Yeast complementation assays, lysate generation and budding indices

Appropriate yeast strains were grown overnight at 25°C, normalized to OD_600_ = 0.1, serially diluted five-fold, spotted onto plates and incubated at 25°C or 32°C for 3 days. For additional details refer to Text S1.

## Supporting Information

Figure S1 **Characterization of SspH2 and HsSGT1 interaction.** The interaction between SspH2 and HsSGT1A was analyzed by co-IP in HEK 293T cells transfected with SspH2 variants (**A**) or HsSGT1A variants and SspH2ΔN'C (**B**). SspH2 and SGT1 constructs were tagged with HA and FLAG epitopes, respectively. IPs and immunoblotting (IB) were performed with the indicated antibodies. These data are summarized in Fig. 1D.
(TIF)

Figure S2 **Comparison of epitope tag position on SspH2 phenotype.** **A,** HEK 293 cells transiently expressing SspH2 carrying epitope tags at the amino- (HA) or carboxyl- (FLAG) terminus, or with HA-SspH1 were metabolically labeled with the palmitate analogue 17-ODYA (100 µM) for 8 h. Incorporation of palmitate analog was detected following click chemistry by streptavidin-Alexa680. Where indicated samples were treated with KOH to confirm the formation of a labile thioester bond between cysteine and palmitate (see Text S1 for details). **B,** HeLa cells were co-transfected with Nod1 and SspH2 carrying epitope tags at the amino- (HA) or carboxyl- (FLAG) terminus, or with empty vector (EV) and treated with Nod1 agonist. Secreted IL-8 levels are presented as the mean ± standard error of the mean for six (EV) and nine (HA-SspH2 and SspH2-FLAG) independent determinations. Data were analyzed using a non-parametric Mann-Whitney test and * denotes *p*<0.05 between the indicated samples and the EV sample. ns denotes no significant difference between the indicated samples.
(TIF)

Figure S3 **Schematic of** ***N. benthamiana*** **Rx-PVX assay.** Two week old *N. benthamiana* plants are silenced for endogenous SGT1 expression using virus induced gene-silencing (VIGS). After three weeks further growth the leaves are infiltrated with *Agrobacterium tumefaciens* harbouring the experimental constructs for transient expression. Following one week of growth the infiltrated leaves are visualized under both UV and brightfield conditions to detect fluorescence and HR lesions, respectively. Lysate is generated from individual leaves and immunoblotted to detect GFP protein levels. Functional Rx-PVX immunity is indicated by an impairment of PVX replication (no detectable GFP protein or GFP fluorescence and possible HR lesion formation). Experimental and relevant control constructs are indicated in the table.
(TIF)

Figure S4 **Characterization of SspH2 enhancement of SGT1-dependent NLR immune response** ***in planta*** **.** **A,** Reciprocal co-immunoprecipitation analyses of AtSGT1B and SspH2 or SspH1 transiently expressed in HEK 293T cells. *S.* Typhimurium effectors and AtSGT1B were tagged with HA and FLAG epitopes, respectively. IPs and immunoblotting (IB) were performed with the indicated antibodies. **B,** Lysates from whole-leaf co-infiltrations of AtA+H2, AtA+H1 and AtA+C580A constructs were immunoblotted (IB) with the indicated antibodies. The membrane was stained with Ponceau S (Pon. S) to indicate protein loading. **C,** Nb Rx TRV:SGT leaf transiently expressing GUS, 5xMyc-AtSGT1A (AtA) and SspH2-3xFLAG (H2) as indicated, were imaged under UV lighting and brightfield 7 days post-infiltration. PVX-GFP was omitted from the assay where indicated (- PVX). Higher magnification panels of the brightfield image are provided for comparison.
(TIF)

Text S1 Supplemental Materials and Methods.
(DOC)
