# Supplementary figures and images for: The Salmonella Type III Effector SspH2 Specifically Exploits the NLR Co-chaperone Activity of SGT1 to Subvert Immunity
Source: PLoS Pathog. 2013 Jul 25;9(7):e1003518. doi: 10.1371/journal.ppat.1003518 (PMC3723637; doi:10.1371/journal.ppat.1003518)

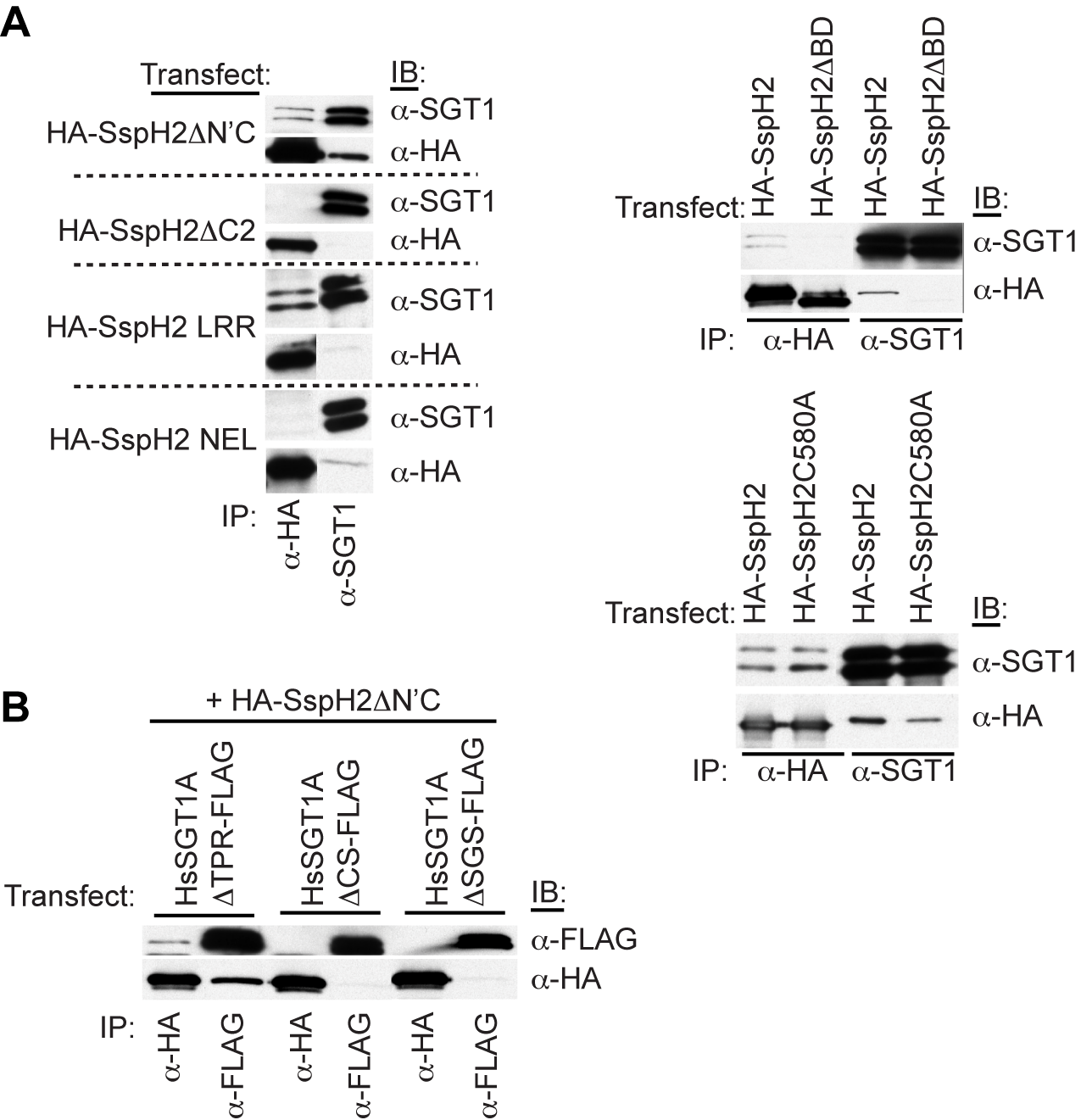

Supplement: Figure S1 — Characterization of SspH2 and HsSGT1 interaction. The interaction between SspH2 and HsSGT1A was analyzed by co-IP in HEK 293T cells transfected with SspH2 variants (A) or HsSGT1A variants and SspH2ΔN'C (B). SspH2 and SGT1 constructs were tagged with HA and FLAG epitopes, respectively. IPs and immunoblotting (IB) were performed with the indicated antibodies. These data are summarized in Fig. 1D. (TIF) [file ppat.1003518.s001.tif]

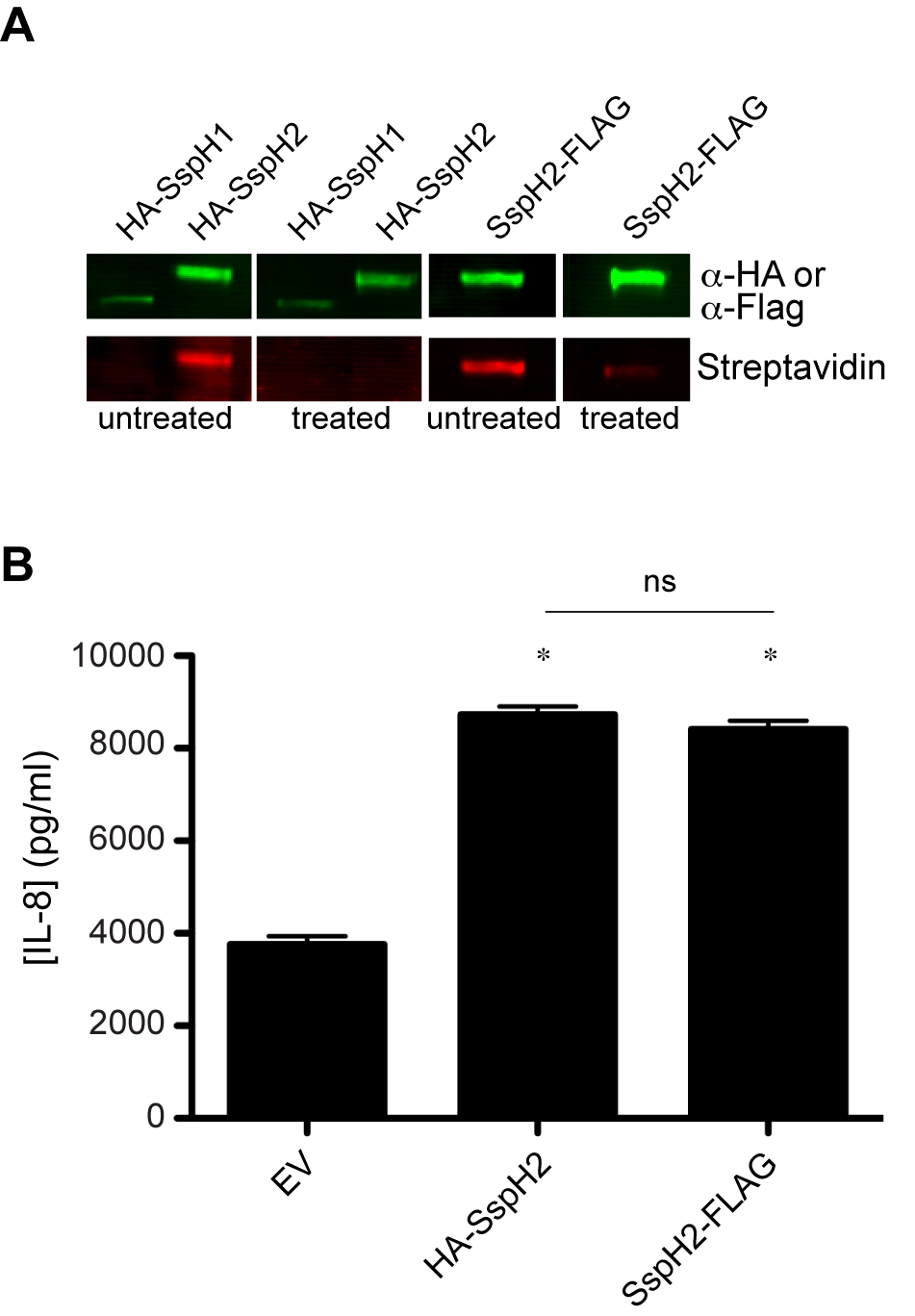

Supplement: Figure S2 — Comparison of epitope tag position on SspH2 phenotype. A, HEK 293 cells transiently expressing SspH2 carrying epitope tags at the amino- (HA) or carboxyl- (FLAG) terminus, or with HA-SspH1 were metabolically labeled with the palmitate analogue 17-ODYA (100 µM) for 8 h. Incorporation of palmitate analog was detected following click chemistry by streptavidin-Alexa680. Where indicated samples were treated with KOH to confirm the formation of a labile thioester bond between cysteine and palmitate (see Text S1 for details). B, HeLa cells were co-transfected with Nod1 and SspH2 carrying epitope tags at the amino- (HA) or carboxyl- (FLAG) terminus, or with empty vector (EV) and treated with Nod1 agonist. Secreted IL-8 levels are presented as the mean ± standard error of the mean for six (EV) and nine (HA-SspH2 and SspH2-FLAG) independent determinations. Data were analyzed using a non-parametric Mann-Whitney test and * denotes p<0.05 between the indicated samples and the EV sample. ns denotes no significant difference between the indicated samples. (TIF) [file ppat.1003518.s002.tif]

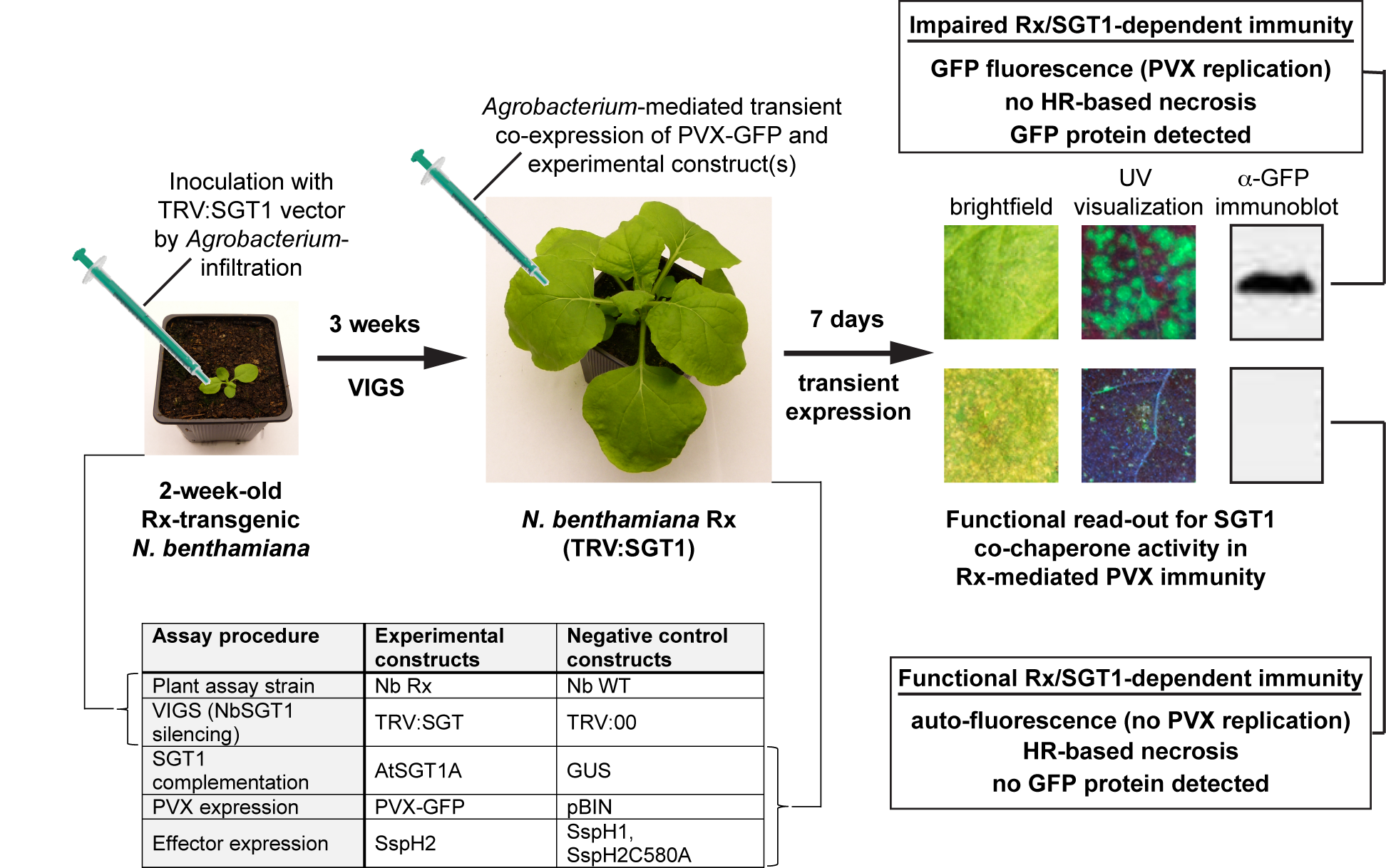

Supplement: Figure S3 — Schematic of N. benthamiana Rx-PVX assay. Two week old N. benthamiana plants are silenced for endogenous SGT1 expression using virus induced gene-silencing (VIGS). After three weeks further growth the leaves are infiltrated with Agrobacterium tumefaciens harbouring the experimental constructs for transient expression. Following one week of growth the infiltrated leaves are visualized under both UV and brightfield conditions to detect fluorescence and HR lesions, respectively. Lysate is generated from individual leaves and immunoblotted to detect GFP protein levels. Functional Rx-PVX immunity is indicated by an impairment of PVX replication (no detectable GFP protein or GFP fluorescence and possible HR lesion formation). Experimental and relevant control constructs are indicated in the table. (TIF) [file ppat.1003518.s003.tif]

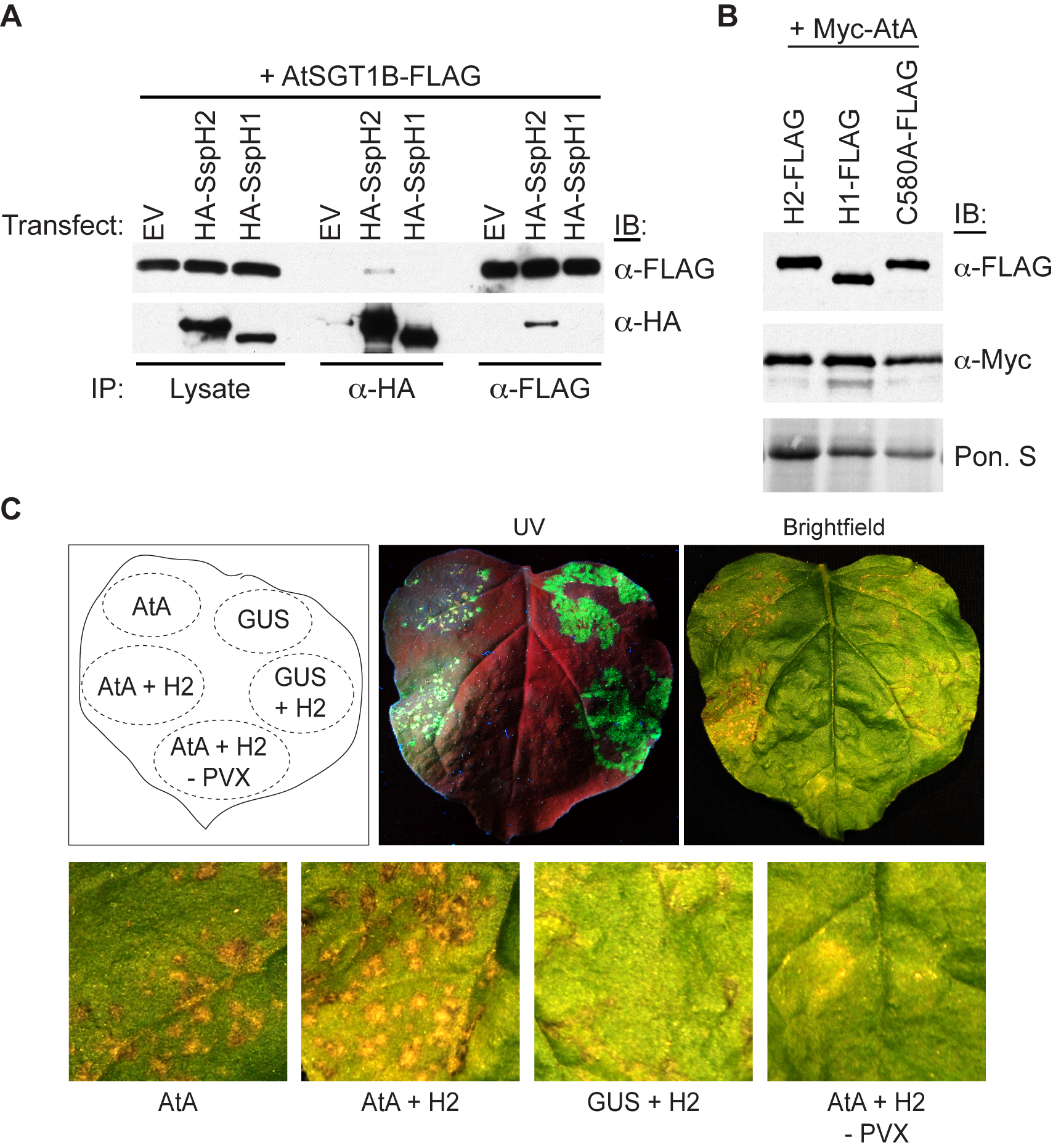

Supplement: Figure S4 — Characterization of SspH2 enhancement of SGT1-dependent NLR immune response in planta . A, Reciprocal co-immunoprecipitation analyses of AtSGT1B and SspH2 or SspH1 transiently expressed in HEK 293T cells. S. Typhimurium effectors and AtSGT1B were tagged with HA and FLAG epitopes, respectively. IPs and immunoblotting (IB) were performed with the indicated antibodies. B, Lysates from whole-leaf co-infiltrations of AtA+H2, AtA+H1 and AtA+C580A constructs were immunoblotted (IB) with the indicated antibodies. The membrane was stained with Ponceau S (Pon. S) to indicate protein loading. C, Nb Rx TRV:SGT leaf transiently expressing GUS, 5xMyc-AtSGT1A (AtA) and SspH2-3xFLAG (H2) as indicated, were imaged under UV lighting and brightfield 7 days post-infiltration. PVX-GFP was omitted from the assay where indicated (- PVX). Higher magnification panels of the brightfield image are provided for comparison. (TIF) [file ppat.1003518.s004.tif]
