# Supplementary material for: The Salmonella Type III Effector SspH2 Specifically Exploits the NLR Co-chaperone Activity of SGT1 to Subvert Immunity
Source: PLoS Pathog. 2013 Jul 25;9(7):e1003518. doi: 10.1371/journal.ppat.1003518 (PMC3723637; doi:10.1371/journal.ppat.1003518)
Supplement: Text S1 — Supplemental Materials and Methods. (DOC) [file ppat.1003518.s005.doc]

**Supplementary Experimental Procedures**

**Cloning** - Truncation variants of *sspH2* were generated by PCR and cloned into pcDNA3.1-2xHA . *sspH2C580A* was generated using Quikchange mutagenesis (Stratagene). *sspH2* was PCR amplified and cloned into pCMV-Tag4A (Stratagene) to create an in-frame carboxyl-terminal FLAG epitope-tagged construct. For complementation of the *S*. Typhimurium *sspH2* deletion strain, *sspH2* together with approximately 1 kb of upstream region was PCR amplified from SL1344 genomic DNA using the primers ssph2xf (5' ATGGTCGACCACAGAATGGCGGCGATATTC 3') and ssph2xr (5' ATGAGATCTGTTACGACGCCACTGAACGTT 3'). The PCR product was cloned into pWSK129-GogB-HA using *Sal*I and *BglI*I restriction enzymes to
replace the *gogB* + upstream sequence with *sspH2* + upstream sequence. The *sspH2* + upstream sequence + HA epitope tags from this plasmid were then subcloned into pWSK29 using *Sac*I and *Kpn*I restriction enzymes to create p*sspH2*-HA.1. *HsSGT1A* and its truncation derivatives were amplified from pTRM4*HsSGT1A* (provided by Katsumi Kitagawa); *AtSGT1A/B* were amplified from pBIN61-5xMyc*AtSGT1A/B* (provided by Ken Shirasu); and *Scsgt1* was amplified from yeast genomic DNA and cloned into pCMV-Tag4A. For *in planta* expression genes were cloned into pBIN61 and expressed from the CaMV 35S promoter with amino-terminal penta-Myc (SGT1 constructs) or carboxy terminal triple FLAG (SspH constructs) epitope tags. *sspH2*, *sspH1* and *sspH2C580A* were directionally cloned into pENTR-D-TOPO (Life Technologies), recombined into pXCSG-*ccdB*-3xFLAG (kindly provided by Jane Parker, MPI, Cologne), re-amplified and sub-cloned into pBIN61. For expression in yeast *sspH2* was recombined into pAG426GAL-*ccdB*-HA and *HsSGT1A/B* and *Scsgt1* were recombined into pAG424GPD-*ccdB*-TAP from their respective entry clones. All PCR-amplified constructs were sequence verified. *AtSGT1A* showed a deviation from the published sequence (C222A) in multiple independent clones although this variant retained biological function. All constructs were propagated in *E. coli* DH10B using standard methods.

**Lysate generation -** For analysis of mammalian cell culture lysate, cells were washed in PBS, lysed in TC Lysis Buffer and centrifuged. For analysis of plant tissue lysate, leaf material was isolated by 6 mm diameter-punch (8-12 total), frozen in liquid nitrogen and homogenized using a tungsten bead in the FastPrep-24 (MP Biomedical). Homogenate was resuspended in SDS-PAGE loading buffer, boiled and centrifuged. For GFP detection, lysate was generated from infiltrations performed on at least three leaves 7 days post-infiltration. For expression controls punches were collected from a whole leaf infiltration 4-5 days post-infiltration. For analysis of yeast lysate, yeast cells were resuspended in TC Lysis Buffer and 0.5 mm glass silica, homogenized in a precellys 24 (Bertin Technologies) and centrifuged.

**Immunoblotting** -Samples were separated on polyacrylamide gels, transferred onto PVDF or nitrocellulose membranes (BioRad), stained with Ponceau S as required and probed with α-HA 3F10 (Roche) from rat; α-HA 12CA5 (Boehringer Mannheim), α-FLAG M2 (Sigma), α-pentaHis (Qiagen), α-Myc 4A6 (Millipore), α-GFP 11E5 (Life Technologies) from mouse; α-FLAG (Sigma), α-calnexin (Enzo Life Sciences) from rabbit; and secondary antibodies α-mouse HRP, α-rat HRP (Jackson ImmunoResearch Laboratories) and α-rabbit HRP (Sigma) from goat. Membranes were developed using ECL Detection Reagent (GE Healthcare) and exposed to BioMax Light Film (Kodak).

**Recombinant protein purification** -Cell lysate of *E. coli* BL21 DE3 containing pDEST17-*HsSGT1A* (provided by Katsumi Kitagawa) was prepared in 20 mM sodium phosphate, pH 7.4, 500 mM NaCl, 25 mM imidazole, 10 μg/mL DNAse, 10 μg/mL RNAse and EDTA-free Complete Protease Inhibitor Cocktail (Roche). Clarified lysate was loaded onto a HisTrap column (GE Healthcare) and eluted with a linear gradient of 20 mM sodium phosphate, pH 7.4, 500 mM NaCl, 500 mM imidazole. Pooled fractions were further purified over S200 and MonoQ columns as outlined in .

**Yeast strain construction** -Wildtype yeast (YPH499 *MATα* *ura3-52 lys2-801 ade2-101 trp1-*Δ*63 his3-*Δ*200 leu2-*Δ*1*) was transformed as described previously using either BPH1123 (pAG426-GAL-*ccdB*-HA) or BPH1124 (pAG426-GAL-*sspH2*-HA). For *sgt1-3* complementation experiments control strains were constructed by transformation of YKK54 with BPH1123 creating YPH2066 (*MATα* *ura3-52 lys2-801 ade2-101 trp1-*Δ*63 his3-*Δ*200 leu2-*Δ*1 sgt1-3:LEU2*, pAG426-GAL-*ccdB*-HA). This strain was separately transformed with BPH1125 (pAG424-GPD-*ccdB*-TAP), BPH1126 (pAG424-GPD-*HsSGT1A*-TAP), BPH1127 (pAG424-GPD-*HsSGT1B*-TAP) or BPH1128 (pAG424-GPD-*Scsgt1*-TAP) creating YPH2067, YPH2068, YPH2069 and YPH2070, respectively. Experimental strains were constructed by transformation of YKK54 with BPH1124 creating YPH2071 (*MATα* *ura3-52 lys2-801 ade2-101 trp1-*Δ*63 his3-*Δ*200 leu2-*Δ*1 sgt1-3:LEU2 ,* pAG426-GAL-*sspH2*-HA). YPH2071 was separately transformed with BPH1125, BPH1126, BPH1127 and BPH1128 creating YPH2072, YPH2073, YPH2074 and YPH2075, respectively.

**Detection of SspH2 palmitoylation** - Preparation and labeling of SspH2 with 17-ODYA was preformed essentially as described previously . Briefly, HEK 293T cells transiently expressing HA-SspH1, HA-SspH2 or SspH2-FLAG were metabolically labeled with 100 µM 17-ODYA for 8 hours in DMEM supplemented with 5% delipidated-FBS and L-glutamine. A labeling period of 8 hours was used because the turnover rate of SspH2 palmitoylation was shown to be relatively slow . Lysis, immunoprecipitation and click chemistry were performed essentially as described previously . Recombinant proteins were immunoprecipitated using mouse anti-HA and mouse anti-FLAG antibodies. Alkyne-palmitoylated proteins were covalently linked to azido-biotin by click chemistry, which was performed off the beads using 1 % SDS in modified RIPA buffer. Following acetone precipitation, the immunoprecipitates were solubilised in 2x Sample loading buffer, separated by SDS-PAGE and transferred to PVDF membranes. Where indicated, membranes were treated with 0.1 M KOH (9:1 methanol:water (v/v)) for 1.5 h at RT to remove palmitoylated proteins. Control membranes were incubated in 0.1 M Tris pH 7.4 (9:1 methanol:water (v/v)). Detection of alkyne palmitoylated proteins was achieved using streptavidin conjugated to Alexa680 (1:2500) and immunoprecipitated proteins were detected using rat-anti-HA (1:2500) and rabbit anti-FLAG. Fluorescent-dye conjugated secondary antibodies were used at 1:2500. Blots were scanned using the Li-Cor Odyssey® Infrared Imaging System.

**Supplementary References**

1. Auweter SD, Bhavsar AP, de Hoog CL, Li Y, Chan YA, et al. (2011) Quantitative mass spectrometry catalogues *Salmonella* pathogenicity island-2 effectors and identifies their cognate host binding partners. J Biol Chem 286: 24023-24035.

2. Coombes BK, Wickham ME, Brown NF, Lemire S, Bossi L, et al. (2005) Genetic and molecular analysis of GogB, a phage-encoded type III-secreted substrate in *Salmonella enterica* serovar typhimurium with autonomous expression from its associated phage. J Mol Biol 348: 817-830.

3. Witte CP, Noel LD, Gielbert J, Parker JE, Romeis T (2004) Rapid one-step protein purification from plant material using the eight-amino acid StrepII epitope. Plant Mol Biol 55: 135-147.

4. Alberti S, Gitler AD, Lindquist S (2007) A suite of Gateway cloning vectors for high-throughput genetic analysis in *Saccharomyces cerevisiae*. Yeast 24: 913-919.

5. Gietz RD, Woods RA (2002) Transformation of yeast by lithium acetate/single-stranded carrier DNA/polyethylene glycol method. Methods Enzymol 350: 87-96.

6. Kitagawa K, Skowyra D, Elledge SJ, Harper JW, Hieter P (1999) SGT1 encodes an essential component of the yeast kinetochore assembly pathway and a novel subunit of the SCF ubiquitin ligase complex. Mol Cell 4: 21-33.

7. Martin BR, Cravatt BF (2009) Large-scale profiling of protein palmitoylation in mammalian cells. Nat Methods 6: 135-138.

8. Yap MC, Kostiuk MA, Martin DD, Perinpanayagam MA, Hak PG, et al. (2010) Rapid and selective detection of fatty acylated proteins using omega-alkynyl-fatty acids and click chemistry. J Lipid Res 51: 1566-1580.

9. Hicks SW, Charron G, Hang HC, Galan JE (2011) Subcellular targeting of *Salmonella* virulence proteins by host-mediated S-palmitoylation. Cell Host Microbe 10: 9-20.
